# A Memoir of Dr. Charles Caldwell

**Published:** 1853-08

**Authors:** L. P. Yandell


Art. II.—*A Memoir of Dr. Charles Caldwell.* By L. P. Yandell, M. D.

Before this article meets the eye of our readers, it is probable that the most distant of them will have heard, through the public prints, of the death of the venerable Dr. Charles Caldwell, so long one of the most frequent contributors to this Journal, and for many years identified with western medical education. The melancholy event took place at his residence, in this city, on Saturday afternoon, the 9th of July.

Dr. Caldwell was not only one of the oldest, but one of the most distinguished men in our profession at the time of his decease. In many respects, he was a very remarkable man. In appearance he was imposing. His bearing and manners in society were striking and peculiar. He attained to an age far beyond the period vouchsafed to the most of men, and a very large proportion of his long life was spent actively in public. He became an author nearly sixty years ago, and few years have passed since without the appearance of something from his pen calculated to keep him before the public mind. Of all the honored names of our profession, no one is, perhaps, more familiar to western medical readers.

Dr. Caldwell was born in Caswell county, North Carolina, of Presbyterian parents, and received the best education that was afforded by that portion of his native

State. At nine years of age he was sent to school, and very soon gave proof of a superior mind. Among those who knew him at this early period and who, having been his school-fellows, retained a lively recollection of his youth, were the late Hon. George W. Campbell, of Tennessee, and Dr. James Blythe, who was for many years before his death his colleague in Transylvania University. They were younger than Dr. Caldwell by several years. They represented him to have been a lad of great energy and ambition, fond of his studies and a proficient in them. From his aspirations and the evidences of mind which he gave, it was easy to be foreseen that he was destined to make a man of note. The profession for which he first studied was Divinity, but at a later period he abandoned it for medicine. Most of his friends have heard him relate the circumstances under which he gave up his original purpose. After quitting the study of theology, he opened a grammar school, in Iredell county, and spent three years as a teacher of boys. He then commenced the study of medicine with Dr. Harris, of Salisbury, and having pursued it privately with that physician for eighteen months, removed to Philadelphia, where he might have the benefit of a public institution. He was in the habit of stating in his lectures, that he continued the study in Philadelphia for five years, and in that time hardly allowed himself one day of relaxation. From the first day that he entered school as a little boy up to a very advanced period of his life, there can be no doubt that he was a most faithful, assiduous student. His account of his habits while a student, and in fact through life, was that he spent from eighteen to twenty hours a day in active mental labor, devoting but four, or at most six hours to sleep.

Soon after graduating he began to publish. One of his first works was a translation of Blumenbach's Physiology, which was followed, some years after, by a translation of Sennac on Fevers. During his pupilage, yellow fever prevailed in Philadelphia, and the origin and nature of the epidemic became the subject of a warm and protracted controversy, in which he took an active part. He contended with Dr. Rush that the disease was of domestic origin, but going farther than his preceptor, insisted that it was not contagious. With these views he urged the futility of quarantine regulations, and the importance of a strict attention to hygienic measures.

Dr. Caldwell manifested thus early a disposition to controversy, which he continued to exhibit all his life. Dr Samuel Stanhope Smith, President of Princeton College, published, in the year 1787, an essay on the "Unity of the Human Race," in which he attempted to explain on physical grounds the diversity of color and physiognomy of the several branches of the human family. Dr. Caldwell, whose studies had been much in the French school popular at that day, attacked Dr. Smith's essay with great boldness, and, as he always himself felt assured, with entire success. But however able his review of the arguments of his theological opponent, the controversy did not bring him the credit which he derived from his discussion of quarantine. On the contrary it subjected him to the suspicion of being unsound in his religious opinions, and created prejudices against him which he did not outlive.

He evinced from the first more taste for the philosophy than the practice of medicine, and was always known rather as a writer than a practitioner. At one time he edited in Philadelphia the Port Folio, and wrote articles on politics, literature, religion, and the current events of the day. He also wrote the biographical sketches of Delaplaine's Repository, and was author of a life of General Green. His addresses before the Philadelphia Medical Society were numerous, and he delivered many political and literary addresses which were published at the time. The subjects upon which his pen was employed, embraced in its wide range, history, poetry, fiction, medicine, hygiene, medical jurisprudence, biography, physics, natural history, belles lettres, and ethnology. He edited Cullen's Practice of Physic, but showed himself most at home when advocating the "vitality of the blood," or attacking Dr. Rush's theory of the "unity of disease." This propensity to generalize—to discuss theories, rather than to multiply facts by engaging in research, gave him early in life a reputation which he never lost—that of being a speculative, visionary man.

His fine elocution naturally suggested to Dr. Caldwell, at an early period, the idea of becoming a public teacher and he was not long in rising to a high rank among the lecturers of Philadelphia. Notwithstanding the hostility of Dr. Rush, which by some means he excited, he always had numerous private pupils, who bore flattering testimony to his eloquence and ability as an instructor. He had become widely known in the Western States as one of the most gifted of the medical writers and teachers in Philadelphia, and when the medical faculty of Transylvania University was reorganized, in 1819, he was elected professor of the Institutes of Medicine. Of the part which he took in building up that school, I have had occasion to speak recently. It is not too much to affirm that he was the father of the first Western School of Medicine. His colleagues were younger men, and were then comparatively unknown. However great the reputation which some of them have since achieved, they did not at first give character to the enterprise, and could not draw to it many students. Dr. Caldwell came to Kentucky a ripe scholar, an experienced teacher, an author of wide celebrity; and it is safe to say that he was now, for the first time, in his proper element. It was in a medical school, as an expounder of the "philosophy of medicine," that he was especially fitted to shine.

His introductory address, delivered at the opening of the first session of the school, was admirably suited to the occasion. It breathed a tone of unbounded confidence. The undertaking, he said, would succeed beyond the expectations of its most sanguine friends, and would confer great and manifold benefits upon the profession and the country. It would throw much new light upon the diseases of the Mississippi valley, and "lead to a more rational and successful treatment of them." By reducing the expenses of a medical education, it would enable persons in moderate circumstances, as well as the wealthy, to attain it. It would promote the study of our climate, and especially its influence in developing or modifying our diseases. And it would "elevate in the west, the standing and dignity of the medical profession, and give it more weight and influence in effecting its benevolent and praiseworthy purposes." The ardor with which he embarked in the enterprise was communicated to his colleagues and to the pupils who had assembled to listen to his inaugural address; and it was only a few years until he had the satisfaction of seeing the school which he had been so instrumental in founding in the backwoods crowded with students, not from the frontier country only, but from Virginia, the Carolinas, Georgia, and other Atlantic States. He devoted himself to the institution. He wrote about it; he traveled to solicit the means for furnishing it with a library and apparatus; he made appeals to the Legislature of Kentucky for an endowment. The money thus raised he applied in Europe himself to the purchase of an excellent library and other means of medical instruction.

It was precisely in such an enterprise as this that Dr. Caldwell's best qualities were most fully and favorably displayed. It brought out that energy and resolution in which he so far surpassed the majority of men. How much soever others might doubt, his confidence was unwavering; whatever might be the faltering of others, his industry was untiring. As a teacher he was not so much esteemed as some of his less distinguished colleagues; but his eloquence, his varied learning, and versatile powers excited the admiration even of those who regarded his lectures as wanting in practical interest. There was an elevation in his style, a measured pomp and roundness in his periods, a clearness in the arrangement of his matter, a confidence of tone and manner in giving out his opinions, a fullness and aptness of illustration in his lectures, which was sure to captivate a part of his audience. In all the classes before which he appeared during his long career as a public teacher, certainly until towards the close of it, there was never one in which there were not a number of students who agreed in placing him at the very head of medical lecturers. The writer of this notice does not hesitate to acknowledge that when a pupil of the Transylvania school he was himself of this number. He can never forget the delight with which he followed there the lectures on the "Institutes of Medicine;" a pleasure which was only exceeded by that of hearing Dr. Holley on Mental Philosophy. An incident occurred during that winter which, as it exemplifies the devotion of some of Dr. Caldwell's pupils to him, and is otherwise illustrative of his character, will not be deemed out of place in this connection.

At the time referred to, Dr. Caldwell took great pleasure in public discussion. He had become a practised and skilful disputant in the Medical Society of Philadelphia, where he had been accustomed to meet Chapman, Cooper, and other adroit debaters. Dr. Drake had just entered the school, and his powers as a controversialist were unknown. He was invited to read a paper before the Lexington Medical Society, at which Dr. Caldwell was present. In the course of his dissertation on certain points in Physiology, Dr. Drake introduced the doctrine of Harvey—*omnia ex ovo;* that all living beings come from germs furnished by parents. The allusion to the doctrine seemed to the audience generally to have been accidental. They could see nothing in it calculated to bring on a long and spirited controversy; but the author of the paper knew full well upon what ground he was entering. He had heard, when a student in Philadelphia several years before, an animated discussion between Dr. Caldwell and Dr. Chapman on the subject of "spontaneous generation," provoked by a paper read to the Medical Society by himself. He did not doubt that Dr. Caldwell was still a believer in that doctrine, and he as little doubted his readiness to support it. Dr. Drake had become aware that he was himself endowed with rare faculties as a debater, and was willing to try his str ngth with his learned colleague. As he expected, soon after he sat down Dr. Caldwell rose and addressed the society. He complimented the paper of Dr. Drake for its ingenuity, learning, and general ability. But there was one tenet, he said, to which he could not subscribe; and he went on to show the grounds upon which spontaneous generation appeared to him still a reasonable hypothesis. Dr. Drake replied, and Dr. Caldwell made a further exposition of the doctrine. It was resolved to continue the discussion at the next meeting of the Society.

It was resumed the ensuing week. In his remarks at the previous meeting, Dr. Caldwell had quoted from the writings of Moses and some of the fathers in medicine certain opinions or statements which seemed to favor the hypothesis he was advocating. This afforded Dr. Drake an apology for bringing forward all the written testimony he could find on the subject, and accordingly he appeared before the society, this evening, with a load of authorities. He piled quotation upon quotation. All the instances cited by his adversary as examples of equivocal generation, he showed, had been accounted for by the presence of germs. It is seldom that one ever listens to a more convincing argument than Dr. Drake made on this occasion. It appeared to his audience conclusive, overwhelming. There was no replying to his facts; no escaping from their irresistible force. The whole medical class, numbering about two hundred, were with him, all but two. One of these was the writer of this sketch; the other was a friend who was also a Tennessean, now no more—George W. Campbell, whose uncle had been the schoolmate of Dr. Caldwell. It is possible that we felt the force of Dr. Drake's powerful argument as much as our classmates, but we could not be made to admit it; we stood resolutely by our favorite teacher. It is not often that a leader has had fewer adherents; but it made no difference with ours, who was impassive to the facts which convinced every body else, and to the last, held as stoutly to his cherished theory as his devoted young friends had done to him.

Dr. Caldwell was as faithful an officer as ever belonged to an institution of learning. He toiled unceasingly for the advancement of the Medical school at Lexington, and it was in the full conviction that its interests could no longer be promoted there, that he advocated its removal to Louisville. He had resisted the conviction as long as he could. After others had begun to talk of erecting a school in this city he wrote in favor of the advantages of Lexington. He defended the site as long as it appeared to him defensible, and with arguments, the soundness of which must have seemed to him questionable while using them. But when he became an advocate for the removal, he was an undisguised, unwavering advocate. He never changed his views, and never deviated from his purpose. He urged the policy openly, and on all proper occasions; and when at length convinced that the Lexington school could not be transferred, he was for establishing a new institution in Louisville.

There is no occasion for adverting in detail to the unpleasant controversy which grew out of the attempt to transfer the Medical department of Transylvania University to Louisville, the history of which is familiar to most of the readers of this memoir. It is well known that Dr. Caldwell bore much more than his share of the censure, attached by the people of Lexington to the medical professors for conceiving and attempting to carry out such a project; and at least one of his colleagues was involved in the acrimonious discussion which followed by his efforts to shield him from the unmerited obloquy. But if he was unduly censured for his interference in behalf of the school at Lexington, he entitled himself, by his zealous efforts to rear an institution in this place, to be called the founder of the University of Louisville. Doubtless the University would have been founded without him, and perhaps founded very soon. The time for erecting a medical school in Louisville had arrived, and many minds were turned favorably towards the measure. But, at the same time, all will admit, that it was Dr. Caldwell who gave the impulse to it at the auspicious moment. He was the leading man in the movement, and his energy and perseverance did much towards securing the donation, and placing the institution upon an ample basis.

Dr. Caldwell was an active and very voluminous writer. He produced no elaborate work, but he was continually writing, now on phrenology, and then on the study of the ancient languages; at one time on mesmerism, and then again on animal chemistry. His pen was never idle. He must write and publish. It had become with him a fixed habit; it was his amusement, his aliment, his life. He was as much the founder of the medical journals as of the medical schools of Kentucky. It was by the dint of his importunity that the medical Faculty at Lexington were induced to project the *Transylvania Journal of Medicine and the Associate Sciences*, and to assure the editors of a supply of matter he pledged himself to contribute to every number. No one had a higher appreciation of the salutary influence of such a periodical upon the profession. He foresaw how it would quicken the medical mind of the country, and what a harvest of experience would be gathered in by it; and it was therefore upon principle that he strove to have one commenced and sustained.—When Louisville became the theatre of his action, he was as zealous for the establishment of a journal here; nor did he rest until he accomplished his wishes. The *Louisville Journal of Medicine* was suspended after the issue of the second number, but he began at once to agitate for a renewal of the effort; and in due season the Journal which is now in the hands of the reader was set on foot. Dr. Caldwell did not edit any of these works, but in one sense they may be said to have originated with him; he was by far the warmest advocate for their projection, and for a long time the most prolific contributor to their pages.

A few years since Dr. Caldwell computed that his writings, up to that time, including translations, pamphlets, essays, and books on various subjects, would amount in the aggregate to more than ten thousand pages, and supposed that they might reach eleven thousand. Since that time he has published probably not less than a hundred pages in the shape of pamphlets and essays. He had then made two hundred and eleven distinct publications, of which six exceeded three hundred pages, thirteen were over two hundred pages in length, twenty-one over a hundred pages; four-fifths of the whole being under fifty pages. It is manifest that it was not by detached, ephemeral tracts, such as most of these, that a solid, enduring reputation was to be achieved. And then the subjects were not only greatly diversified—too numerous, it may be affirmed, to be thoroughly compassed by any human intellect; but many of them were unfortunate subjects Too many of them were topics that serious, philosophical minds have agreed in rejecting as unworthy of grave investigation; and upon too many more the opinions which he entertained were at war with the science of the age. The greatest of his works, unquestionably, are the schools which he founded. Aside from his merely literary productions and his translations, much the larger portion of his writings relates to animal chemistry, phrenology, and mesmerism. When he touched upon questions pertaining to natural theology, he continually broached views which gave offence to the friends of revelation. On the physiological topics, about which he wrote most copiously—'solidism,' the 'humoral pathology,' 'sympathy,' animal chemistry'—he stood almost alone, vainly struggling against the onward progress of scientific discovery. Phrenology, and mesmerism, the cherished topics upon which he bestowed most time and labor, and which it was his glory to have been foremost in advancing, whatever may be their real merits, have not yet obtained a footing among the accredited sciences. It seems to have been a peculiarity of the mental constitution of this gifted man, that the truths which most thoughtful men regard as essential and best established, were those about which he doubted most, while the doctrines which scientific men generally condemn, were those which he most cordially embraced. The effect upon his reputation was unhappy while he lived, and cannot fail to be so upon the popularity of his writings.

His style, when young, was involved, inflated, diffuse, and inclined to bombast. In these respects it improved with age, without ever becoming easy, simple, or graceful; but it always possessed the qualities of vigor and clearness, and had the merit also of being peculiar. No one was ever at a loss for his meaning, or could be long in doubt as to his authorship. His remarkably fine personal appearance and bearing were not more distinctive than certain forms of expression which were sure to reappear in his writings and at once revealed their author. No author, perhaps, ever repeated himself so often; and this gave a monotony to his productions, especially the later ones, which detracted very much from their interest.

The subject being given, it was easy for those who were familiar with his writings, to anticipate, not the drift and arrangement of his discourse merely, but the facts, the arguments, the illustrations, and even many of the very phrases. In conversation, in writing, and in lecturing he was the same; always forcible, earnest, and confident, generally elevated, never obscure. His mind was always full of his subject, and he never failed to make himself understood by his readers or hearers of whatever capacity. If he failed often to carry conviction to others, it was not because he had not the air of being himself fully persuaded of the truth of what he urged.

Of all his professional cotemporaries in this country, Dr. Caldwell had the highest opinion of the talents of the late Dr. Chapman. No medical man in America was deemed by him to possess so inventive and original a mind. There is something interesting in the circumstance, that these eminent men, who had lived as friends so long in the same city, and pursued together the same line of investigation, should have closed their earthly career so nearly at the same time. In the mould and texture of their minds, and in their general bearing, Dr. Caldwell and Dr. Chapman were not unlike each other. They were both finished scholars, and ornate, showy lecturers; they adopted a similar style of writing and declamation; their tastes inclined them to the same course of study, and they embraced many of the same unpopular doctrines in medicine. They were alike exclusive and extravigant in their views concerning the humoral pathology, sympathy, and the vital principle. The notions which Dr. Chapman early in life put forth in respect to the *modus operandi* of medicines, Dr. Caldwell was found strenuously supporting in some of the last papers he ever published. Dr. Chapman was the more practical man of the two, and being drawn by circumstances more towards the active duties of his profession, was saved from those purely speculative and visionary studies upon which his friend wasted so much of the vigor of his life. They rose to almost equal distinction. One was at the head of his profession at the East; the other was for a long time the acknowledged head in the West. They were both connected for many years with flourishing medical schools. Dr. Caldwell had not the readiness or the wit of Dr. Chapman, but he had more energy, and greater powers of application; he was better fitted for rearing an institution of learning in a new country. In both were united to minds of a high order talents for display which gave them great influence among men.

Among the associates of Dr. Caldwell in his long connection with the medical schools of Kentucky, the colleague whom he ranked highest for originality and vigor of mind was Dr. Drake, who also has preceded him to the grave. Dr. Drake always claimed to have been the the pupil of Dr. Caldwell, having attended his private lectures in Philadelphia, and was a younger man by a good many years. Their association in important enterprises during the most active period of their lives naturally suggests a comparison of the characters of these distinguished men. There were few points of resemblance between them. In manners, tastes, style of writing and speaking, habits of thought, social impulses, and medical and philosophical opinions, they differed the most widely from each other. Except minds of great compass, rare strength of will and energy of purpose, and an industry untiring in both, they possessed few qualities in common. Dr. Caldwell had the advantage of a s superior education, but Dr. Drake continued to be the greater reader, and so kept himself abreast of the tide of discovery in his profession and conformed his teachings to it. Dr. Caldwell had great boldness, and launched his theories forth regardless of the opinions of other men. "Better occasionally broach startling errors than deal in time-beaten truisms which awaken no curiosity," was one of his published sentiments upon which he acted.—Dr. Drake was cautious in hazarding opinions which he formed after long investigation. His style of speaking was less finished and graceful than that of Dr. Caldwell, but it was more earnest and effective. His voice was more powerful, and the operation of his mind far more rapid and intense. He had a readiness, a keenness, a command of language and apt illustration, that rendered him formidable in debate, and quite an over-match for his more learned colleague. In manner and appearance one of the most diffident of men, when he engaged in a controversy he was one of the most assured. His wit, his readiness of repartee, his acuteness of analysis, delighted and astonished his audience. Dr. Caldwell excelled in a clear exposition of his subject; in the methodical arrangement of his positions; in the symmetry and order of his discourses, where there were none to controvert or object. Dr. Drake excelled in debate in which his wonderfully active mind was always ready with the ingenious argument, the pertinent fact, the happy retort. His style of writing was more simple and natural than Dr. Caldwell's, and therefore a better style for scientific subjects. In conversation, the difference between them was that Dr. Drake was unequal—sometimes dull and tiresome; sometimes singularly eloquent, original, brilliant, and instructive; while Dr. Caldwell always did justice to his reputation. In personal appearance Dr. Caldwell had the advantage. His head was one for the sculptor, as classical, as finely developed as phrenologist could desire to behold. His person was large, well-proportioned, stately and commanding. No one could see him without remarking him.

It has been remarked, that Dr. Caldwell's taste led him to the speculative in science. Dr. Drake inclined to the practical. When a young man, Dr. Caldwell gave lectures on natural history, but it was the natural history of books; he made no observations of his own; engaged in no original researches; and later in life he forsook these studies for psychology. Dr. Drake was always an observer, and from the earliest period to the close of his professional life was engaged in independent research. His first work—the *Picture of Cincinnati*—was a volume of facts and statistics; his last and great work—the *Diseases of the Interior Valley of North America*—was like it, a vast treasury of facts gathered up through numerous years and in the course of many long journeys. While Dr. Caldwell showed more perseverance in erecting medical schools, Dr. Drake showed greater steadiness of literary aim; and if the latter left behind him no successful institution to which he could point as of his creation, he produced a work of which Dr. Caldwell, among all his publications, has left behind him no equal.

Dr. Caldwell's excellence as a medical teacher lay chiefly in the faculty of inspiring his pupils with a love of science, and an ambition to excel, and in this he was eminent. His defects were those that belong to his writings;—a propensity to speculate; an undue preference for subjects of equivocal value; a proclivity to doctrines generally discarded. A more serious defect, which, whether it was engendered by the prejudices of early education or by associations formed later in life, is lamented by his friends as the capital one of his character, was the want of a sincere religious faith. This showed itself in whatever he did. It was continually appearing in his writings; it was remarked in his daily conversations; and he was unable to conceal it in his public lectures. It was keenly felt by himself; no one, probably, knows how keenly. "*I keep up a storm without to silence the storm with-*

*in,*" he has been heard to remark of himself. He was aware how much of influence he had lost from this cause; but he could not have been aware how much it had to do in giving his mind those unfortunate scientific biases which the friends of his fame have to regret. With a mind more happily constituted in this respect, possessing a person so distinguished, talents so varied and so highly cultivated, so fine an elocution, such physical powers of endurance, and an energy so indomitable, it would be difficult to say to what height he might not have risen.

